# The genus *Gennadas* (Benthesicymidae: Decapoda): morphology of copulatory characters, phylogeny and coevolution of genital structures

**DOI:** 10.1098/rsos.171288

**Published:** 2017-12-06

**Authors:** Alexander L. Vereshchaka, Anastasia A. Lunina, Jørgen Olesen

**Affiliations:** 1 Shirshov Institute of Oceanology, Russian Academy of Sciences, Nakhimov Prospekt 36, Moscow 117997, Russia; 2 Natural History Museum of Denmark, University of Copenhagen, Universitetsparken 15, 2100 Copenhagen, Denmark

**Keywords:** shrimps, *Gennadas*, Benthesicymidae, phylogeny, evolution, copulatory structures

## Abstract

Species within *Gennadas* differ from each other largely only in male (petasma) and female (thelycum) copulatory characters, which were restudied in scanning electron microscopy and used as a basis for phylogenetic analyses. Twenty-six petasma characters and 49 thelycum characters were identified. All 16 recognized species of *Gennadas* and *Aristaeomorpha foliacea* (outgroup) were included as terminals. Four robust monophyletic clades were retrieved, described and diagnosed as new species groups. The thelycum characters had greater impact on tree topology and supported deeper nodes than did the petasma characters. We hypothesize that features of the thelycum evolved first followed by aspects of the petasma. Relatively more conservative characters include parts of the sternites of the thelycum and of the petasma, while the scuti and protuberances on the thelycum and the shape and subdivisions of the petasma lobes are evolutionarily plastic. We identified two groups of copulatory characters, which are likely coupled functionally and interlinked evolutionarily: (i) the external part of the petasma and the posterior part of the thelycum and (ii) the internal part of the petasma and anterior part of the thelycum. We reconstruct possible mating position during copulation for each of the new species groups presented here. We also present an updated key to genera of Benthesicymidae and key to species of *Gennadas*.

## 1. Introduction

The family Benthesicymidae includes 40 valid species in five genera: *Altelatipes*, *Bentheogennema*, *Benthesicymus*, *Benthonectes* and *Gennadas* [1,2]. The family remains underexplored taxonomically, partly because the genus occurs in the deep sea. Indeed, four of five generic names start either with Benthe (from Greek benthos, ‘depth of the sea’) or Alte (altus, ‘deep’). Owing to deep habitat preserved material is often in poor condition and many species are represented by a restricted number of individuals. Finally, Benthesicymidae are very similar in general morphology within the genera and only copulatory structures allow confident identification to species level.

This paper is devoted to the genus *Gennadas*, the most abundant and diverse genus of Benthesicymidae, which encompasses 16 of the 40 species of the family (40%). Most species of *Gennadas* are abundant and widely distributed in all oceans and therefore were discovered and described already in the late nineteenth century or in the beginning of twentieth century. Only one species, associated with the seamounts of the distant Nazca and Sala-y-Gomez Ridges, was described later (*Gennadas barbari*: Vereshchaka, 1990 [3]). Burkenroad [4], Tirmizi [5] and Kensley [6] described and figured a number of species of *Gennadas*, but made no attempt to revise the systematics of the genus on a global scale. Moreover, there are some ambiguities regarding some of the species in the most recent species list of the genus [2]. For example, *Gennadas crassus* Tirmizi, 1960 [5] is based only on a single female and no males have since been reported; *Bentheogennema burkenroadi* Krygier and Wasmer, 1975 [7] has, despite having greatly elaborate copulatory structures similar to those in *Gennadas*, until now not been placed in this genus.

In addition to the general biodiversity and taxonomic interest related to *Gennadas*, the genus offers a number of phylogenetic challenges due to the general similar morphology of many species. Indeed, regarding the external morphology, the genus is morphologically more uniform than the rest of the Benthesicymidae, which may be explained by the fact that they occupy very similar ecological niches in the marine habitats: all species are mesopelagic migrants occurring between 500 and 1500 m in the daytime and between 200 and 500 m at night in the Atlantic [8], Pacific [3] and Indian [9,10] Oceans.

However, genital structures (female thelyca and male petasmata) show an outstanding diversity in *Gennadas* and are therefore promising for phylogenetic reconstruction as was shown for other Dendrobranchiata [11–14]. Despite a greatly elaborate morphology, structural variation in the thelyca and petasmata between specimens are well known to be negligible, so these organs have traditionally been used as ‘fingerprints’ for species identification [3,5,10].

In this study, we provide an inventory of the global fauna of *Gennadas*. We also restudy and/or revise all available copulatory structures in search of suitable phylogenetic characters on which a new classification can be based. As in previous studies on the classification of other pelagic shrimps, we examine genital structures by the use of scanning electron microscopy (SEM). Based on the phylogeny, we test statistically the possibility of coevolution between male and female genital structures, test their contribution to the phylogeny and discuss questions such as: which genital structures arose first, female or male? Which genital structures are evolutionarily conservative and which are more plastic? Based on both phylogenetic and morphological considerations, we also propose hypotheses for how the complex female and male genital structures may operate and interact with each other during spermatophore deposition.

## 2. Material and methods

### 2.1. Material and morphological analysis

The material used for this study is primarily from Danish and Russian expeditions exploring the pelagic zone of the world oceans. Most material used for the study is stored in the crustacean collection of the Natural History Museum of Denmark (NHMD) (electronic supplementary material, appendix S1). The work involved sorting and identification of the about 200 samples of *Gennadas*; individuals were then selected for further study by SEM.

**Figure 1.**
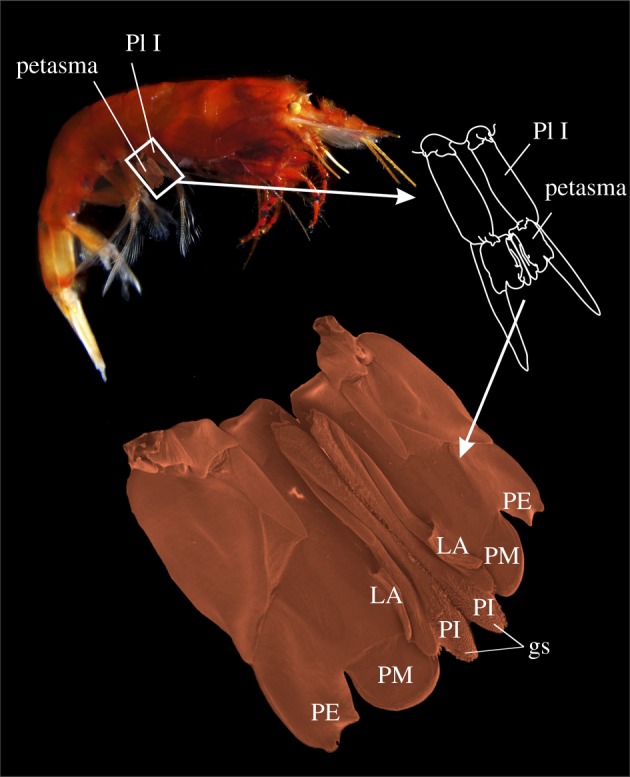
Position and structure of the complete petasma in *Gennadas*, both symmetrical halves included. Abbreviations: PI, pars interna; PM, pars media; PE, pars externa; LA, lobus accessorius; gs, grasping structure; Pl I, first pleopod.

**Figure 2.**
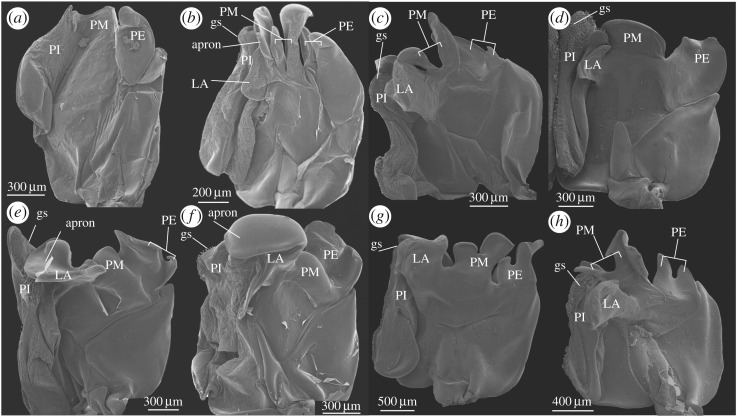
Petasmata of *G. elegans* (*a*), *G. barbari* (*b*), *G. gilchristi* (*c*), *G. tinayrei* (*d*), *G. parvus* (*e*), *G. sordidus* (*f*), *G. valens* (*g*) and *G. talismani* (*h*). Right halves (petasmata symmetrical), anterior view (side faced to the abdomen in dead specimens). Abbreviations: PI, pars interna; PM, pars media; PE, pars externa; LA, lobus accessorius; gs, grasping structure.

**Figure 3.**
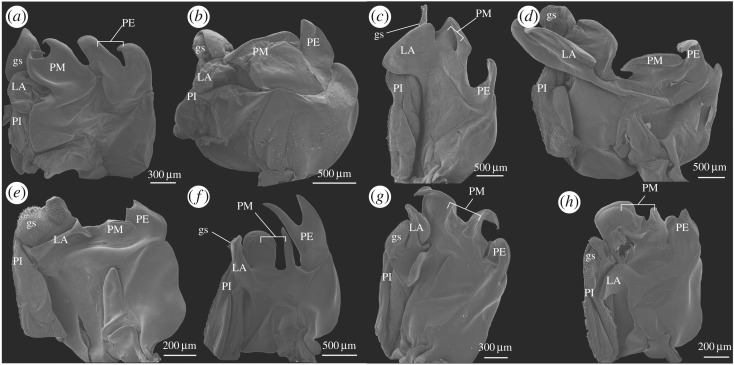
Petasmata of *G. bouvieri* (*a*), *G. kempi* (*b*), *G. brevirostris* (*c*), *G. burkenroadi* (*d*), *G. capensis* (*e*), *G. incertus* (*f*), *G. propinquus* (*g*) and *G. scutatus* (*h*). Right halves (petasmata symmetrical), anterior view (side faced to the abdomen in dead specimens). Abbreviations: PI, pars interna; PM, pars media; PE, pars externa;LA, lobus accessorius; gs, grasping structure.

**Figure 4.**
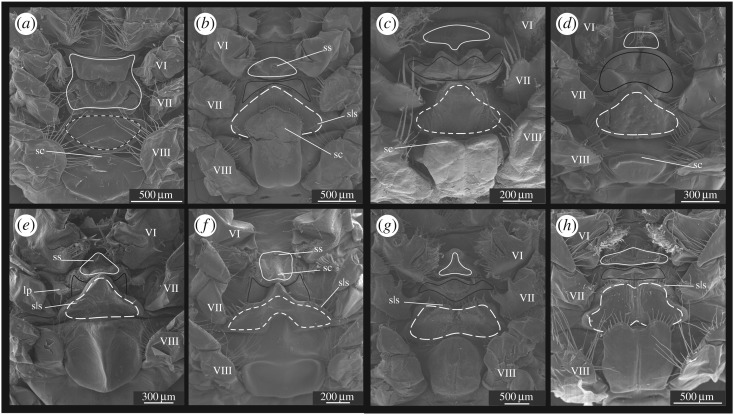
Thelyca of *G. elegans* (*a*), *G. barbari* (*b*), *G. gilchristi* (*c*), *G. tinayrei* (*d*), *G. parvus* (*e*), *G. sordidus* (*f*), *G. valens* (*g*) and *G. talismani* (*h*). Outlines: solid white (sixth thoracic sternite), solid black (anterior part of seventh thoracic sternite) and white dash (posterior part of seventh thoracic sternite). Latin numbers indicate coxae of respective thoracic segments. Abbreviations: sc, scutum; sls, sublateral setae; ss, specialized setae.

**Figure 5.**
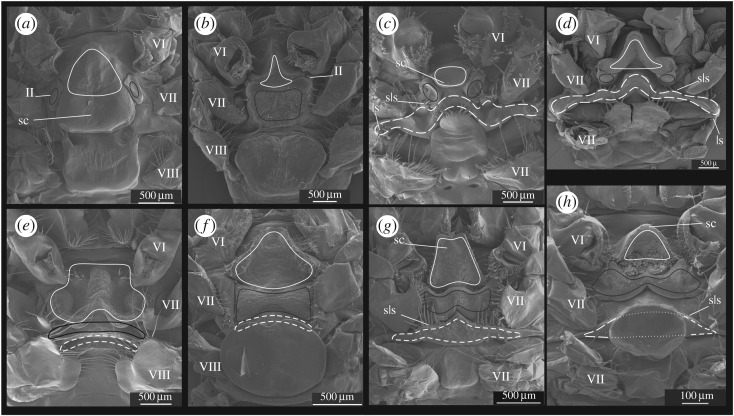
Thelyca of *G. bouvieri* (*a*), *G. kempi* (*b*), *G. brevirostris* (*c*), *G. burkenroadi* (*d*), *G. capensis* (*e*), *G. incertus* (*f*), *G. propinquus* (*g*) and *G. scutatus* (*h*). Outlines: solid white (sixth thoracic sternite), solid black (anterior part of seventh thoracic sternite) and white dash (posterior part of seventh thoracic sternite). Latin numbers indicate coxae of respective thoracic segments. Abbreviations: sc, scutum; sls, sublateral setae; ls, lateral setae.

Prior to treatment for SEM, relevant parts (such as the thelycum, the petasma and the appendix masculina) of selected specimens were dissected in order to expose important structures for further study. The material was dehydrated in a graded ethanol series, critical point dried, mounted and coated with a mixture of platinum and palladium following standard procedures [15]. The SEM used was a JEOL JSM-6335F (with a field emission gun). The images were processed and arranged in standard graphical software such as CorelDraw X7 and various Adobe programs. The position and structure of the complete *Gennadas* petasma, which consists of two symmetrical halves, are represented in figure 1. Petasmata and thelyca of all valid species are represented in figures 2,3 and 4,5, respectively.

### 2.2. Phylogenetic analyses

Since the taxonomic validity of the genera of Benthesicymidae are uncertain and the phylogenetic relationships between them unknown, an outgroup was chosen outside the family. As molecular data have shown, Benthesicymidae is sister to Aristeidae [16] and we chose *Aristaeomorpha foliacea* (Risso, 1827) [17], type for genus by original designation, as the outgroup. All valid species of *Gennadas* and *Bentheogennema* were included as terminal taxa.

We used 49 thelycum-related and 26 petasma-related characters (electronic supplementary material, appendix S2). The data matrix is presented in electronic supplementary material, appendix S3. To estimate the contribution of the female and the male genital structures to the morphology-based phylogeny of *Gennadas*, we made three analyses: (i) one with only female characters included (Analysis 1), (ii) another with only male characters included (Analysis 2) and (iii) a final with all characters included (‘total evidence’, Analysis 3). Data were handled and analysed under maximum-parsimony settings using a combination of programs: WINCLADA/NONA, NDE (Nexus Data Editor) and TNT [18,19].

All characters were unordered (non-additive) and equally weighted. Trees were generated in TNT using the ‘implicit enumeration’ options. Relative stability of clades was assessed by standard bootstrapping (sample with replacement) with 10 000 pseudoreplicates and by Bremer support (algorithm tree bisection–reconnection, saving up to 10 000 trees up to eight steps longer). We considered the clades statistically significant if they were supported both by Bremer values greater than or equal to 3 and bootstrap values greater than or equal to 80.

### 2.3. Assessment of possible coevolution between copulatory structures

To assess possible coevolution of characters, all morphological characters were divided into nine groups associated with the following structures (see electronic supplementary material, appendix S2 for character scoring): (i) sixth thoracic sternite in females (characters 1–9), (ii) anterior part of the seventh thoracic sternite in females (characters 10–25), (iii) posterior part of the seventh thoracic sternite in females (characters 26–39), (iv) eighth thoracic sternite in females (characters 40–48), (v) pars interna (PI) of the petasma (‘internal lobe’ in [1]: characters 49–50), (vi) lobus accessorius (LA) of the petasma (characters 51–56), (vii) pars media (PM) of the petasma (‘median lobe’ in [1]: characters 57–64) and (viii) pars externa (PE) of the petasma (‘lateral lobe’ in [1]: characters 65–74).

In the resulting ‘total evidence’ phylogenetic tree, we considered each node as an evolutionary event and calculated how many character states within each group synchronously changed at each node of the tree. Changes in the character states at each node were considered as binary parameters (absent or present) and further analysed via ANOSIM and non-metric MDS analysis and hierarchical clustering (single linkage algorithm and Kulzcynsky similarity index).

Calculations and analyses were carried out with the use of Excel, STATISTICA and PAST v. 3.04 [20]. Correlations were considered significant if *p* < 0.05.

## 3. Results

### 3.1. Ultrastructure of the thelycum

The thelycum in *Gennadas* is formed by the sixth (S6), seventh (S7) and eight (S8) thoracic sternites. The seventh sternite is split into an anterior (AS7) and a posterior (PS7) parts in all *Gennadas* species, except *G. elegans* (figure 4*a*). The shape of each sternite is very characteristic in the various species and a number of distinct types could be recognized as seen in the following.

S6 is small and subtriangular in all *Gennadas*, except *G. elegans*, in which S6 is enlarged and has a complex relief (figure 4*a*).
AS7 may be simple and unspecialized, without prominent relief (figure 5*a*,*b*,*e*,*f*), trapezoid with posterior incision (figure 4*b*,*e*,*f*), bilobed with notched elevation (figure 4*c*,*d*), bat-like with lateral edges not reaching coxae (figure 4*g*,*h*), as two separate lateral ear-like structures (figure 5*c*,*d*), or W-shaped with lateral edges reaching coxae (figure 5*g*,*h*).
PS7 may be simple and unspecialized, without prominent relief (figure 5*a*,*b*,*e*,*f*), subtriangular (figure 4*b–f*), trapezoid, with anterior incision (figure 4*g*,*h*), as a long chitinized strip laterally produced beyond coxae (figure 5*c*,*d*,*g*,*h*).
S8 are nearly ortho-/pentagonal or trapezoid, sometimes soft and not chitinized (figure 5*e*), may have medial ridges (figure 4*e,f*), medial grooves (figure 4*g*,*h*) or antelateral spinose extensions (figures 4*h* and 5*a*).

Each of sternites may have a scutum of various size, shape, direction and armature. The scutum-related characters are very characteristic for each species (figures 4 and 5). In some cases, scuti are greatly expanded and cover main part of the thelycum, either being posteriorly produced from S6 (figure 5*a*) or anteriorly produced from AS7 (figures 4*b* and 5*h*). In *G. incertus* (figure 5*f*), a very unusual structure is produced from the central part of S8 and extending in all directions as a mushroom cap over S8.

In addition to scuti, species of *Gennadas* (all except *G. elegans*) have strong specialized setae of various size and shape. They may include medial setae on S6 (figure 4*b*,*e*,*d*), sublateral and lateral setae (figure 5*c*,*d*) on PS7.

The seminal receptacles in *Gennadas* are usually present (closed thelycum), although they may be developed to different extent. The female orifices may be guarded by scuti, which are either posteriorly extended from S6 (figures 4*a*,*b*,*e*,*f* and 5*a*,*e*) and/or anteriorly extended from AS7 (figures 4*c*,*e*,*f*,*h* and 5*b*). In four species, orifices are bordered by compound posterior and anterior projections of S6 and AS7, respectively (figure 5*c*,*d*,*g*,*h*). In *G. tinayrei*, the receptacles involve also fifth thoracic sternite (figure 4*d*), which have a long posteriorly directed spinose process.

The description of *G. crassus* Tirmizi, 1960 [5] was based on a single female individual and no records of either female or male have been made since. We have examined the holotype (focusing on the thelycum) and found no difference between this and the thelycum of *G. gilchristi*. Taking into account the very elaborate thelyca in all other *Gennadas*, which are used as a fingerprint for identification of species, we consider the former species a junior synonym of the latter.

### 3.2. Ultrastructure of the petasma

The petasma is consisting of PI, PM, PE and LA in all species except *G. elegans*, in which PM and LA are absent (figure 2*a*).

PI is covered with one to five rows of circinnuli along the medial edge. In all species except *G. elegans* (figure 2*a*), the distal part of PI is transformed into what we propose is a specialized grasping structure, which is characteristic for each species and may include protuberances and invaginations of various sizes and shapes (figures 2*b–h* and 3*a–h*). The grasping structure is well chitinized and rigid in most species, soft and folded in two species (figure 3*a*,*b*). Sometimes, the grasping structure bears circinnuli greatly enlarged and/or transformed into hooks and/or spines (figures 2*d*,*f* and 3*e*,*h*).

LA is extending from that side of the petasma, which is directed towards the abdominal sternites. In some species, LA is very characteristic and has a greatly expanded apron-like apex (figure 2*e*,*f*) or terminal hook (figure 3*d*). LA may be as a club, which is nearly orthogonal to the main lamella (figure 2*h*) or flat and laterally extended, overlapping PI in the lateral direction (figure 3*c*,*d*).

PM may be short and rounded (not reaching the end of PI and PE; figure 2*e*,*f*) or wide and long (reaching the end of PI and PE; figure 3*a*,*b*) lobe. PM may consist of two independent lobules, which are either joining basally (figures 2*g*,*h* and 3*g*,*h*) or widely separated (figure 3*f*).

PE may be entire (figures 2*a* and 3*b*) or consisting of two long independent lobules (figures 2*b* and 3*a*). PE may have a terminal notch dividing apex either into pointed (figure 3*e*,*f*) or rounded (figure 2*g*,*h*) branches. PE may have an additional, small, rounded (figure 3*d*), pointed (figure 2*e*,*f*) or toothed (figure 2*c*,*d*) lobule. PE may have a few terminal tiny teeth (figure 2*d*) or be serrate (figure 3*d*).

### 3.3. Phylogenetic analyses

**Figure 6.**
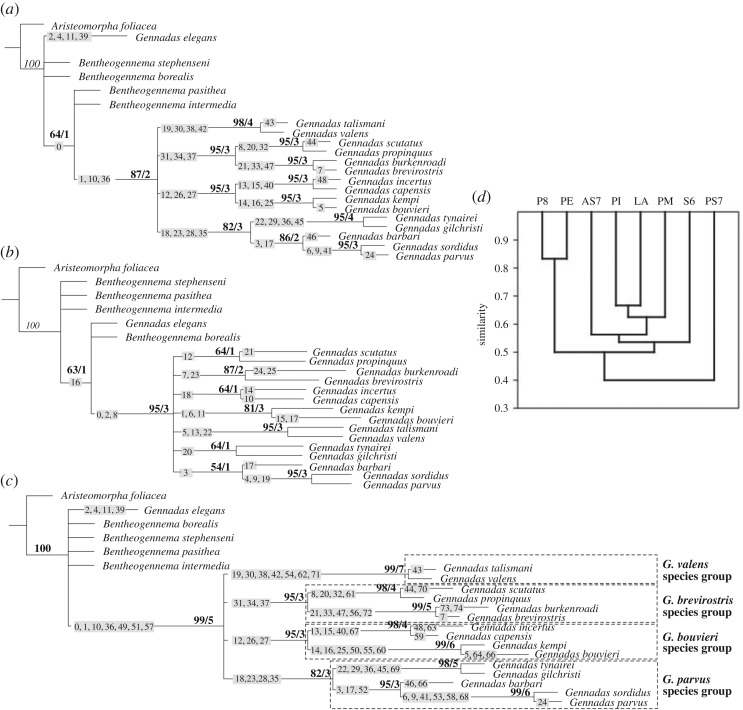
Phylogenetic MP trees of *Gennadas* with *A. foliacea* as outgroup: with only thelycum-related characters included (*a*), with only petasma-related characters included (*b*), with all characters included (*c*); hierarchical clustering of evolutionary events linked to copulatory structures (single linkage algorithm and Kulzcynsky similarity index) (*d*). Numbers on clades refer to synapomorphies (electronic supplementary material, appendix S2), numbers above clades refer to bootstrap (before slash) and Bremer (after slash) support. Abbreviations: S6, sternite of the sixth thoracic segment; AS7 and PS7, anterior and posterior parts of sternite of the seventh thoracic segment; S8, sternite of the eighth thoracic segment; PI, pars interna of the petasma; PM, pars media of the petasma; PE, pars externa of the petasma; LA, lobus accessorius of the petasma.

Analysis 1 (figure 6*a*) with only thelycum-related characters retrieved a single most parsimonious (MP) tree, with a score of 50 (CI = 98, RI = 98). The trees showed that *G. elegans* (along with all *Bentheogennema* species) was separate from the core *Gennadas*, which further branched into four clades (figure 6*a*): (i) *G. talismani* + *G. valens*, (ii) *G. scutatus* + *G. propinquus* + *G. burkenroadi* + *G. brevirostris*, (iii) *G. incertus* + *G. capensis* + *G. kempi* + *G. bouvieri* and (iv) *G. tinayrei* + *G. gilchristi* + *G. barbari* +*G. sordidus* *+* *G. parvus.* The last three clades were further divided into six terminal clades, consisting of two species each. All these clades received high statistical support.

Analysis 2 (figure 6*b*) with only petasma-related characters retrieved a single MP tree, with a score of 27 (CI = 96, RI = 95). The trees showed a basal branching similar to that of Analysis 1 (*G. elegans* outside the remaining *Gennadas*), while further branching was greatly less resolved. Only terminal clades (same pairs of species as in Analysis 1) were revealed, but only three were robust: *G. kempi* + *G. bouvieri*, *G. talismani* + *G. valens* and *G. sordidus* *+* *G. parvus*.

Analysis 3 (figure 6*c*) with all characters included retrieved a single MP tree, with a score of 80 (CI = 96, RI = 97). The trees had a topology very similar to that of Analysis 1. All clades received better support (figure 6*c*). As in Analysis 1, the branching can be described as occurring at three levels: (i) basal branching (*G. elegans* and the core *Gennadas*), (ii) medial-level branching into four species groups and (iii) terminal branching with each terminal clade consisting of two species.

### 3.4. Synapomorphies

The core *Gennadas* (all species except *G. elegans*) was supported by synapomorphies mostly involving general morphological characters: presence of specialized setae, shape/division of thoracic sternites in the thelycum and presence/absence of lobes in the petasma (figure 6*c*). The medial-level branching was supported by synapomorphies mostly relating to the morphology of the medial part of the thelycum (AS7 and PS7); *G. valens* species group was additionally supported by characters relating to S8 and the petasma (figure 6*c*). The terminal clades (each consisting of two species) were supported by the characters related to detailed morphology: degree of extension and chitinization of sternites, presence and shape of specialized groups of setae, shape and development of scuti on the thelycum and lobi on the petasma (figure 6*c*).

### 3.5. Coevolution of morphological characters

ANOSIM multidimensional scaling and hierarchical clustering showed that morphology of different parts of copulatory structures have coevolved (figure 6*d*). The use of all available evolutionary events (nodes on phylogenetic tree—figure 6*c*) showed the following correlations:

(1) between posterior part of the thelycum (S8) and external part of the petasma (PE) and
(2) between anterior part of the thelycum (S6 and AS7) and interior part of the petasma (PI, PM and LA).

One part of the thelycum (PS7) was not grouped with any of the other copulatory structures.

## 4. Discussion

### 4.1. Taxonomic implications

#### 4.1.1. The status of *Bentheogennema burkenroadi*

*Gennadas burkenroadi* was described as *Bentheogennema burkenroadi* Krygier and Wasmer, 1975 [7]. The authors noted that this species has an intermediate position between *Gennadas* and *Bentheogennema* in having a single pair of lateral spines on the telson (as in *Gennadas*, *Bentheogennema* has two to four pairs) and podobranchs on the second maxilliped posteriad to the third pereopod (as in *Bentheogennema*, *Gennadas* has podobranchs on the second maxilliped only). The authors considered podobranchs as a ‘primitive characteristic, a more important generic trait than the number of pairs of spines on the telson’ [7, p. 49] and put the new species into *Bentheogennema*. We consider both traits above as equally important in the generic taxonomy of Decapoda and therefore refer to two additional evidences to resolve the position of *B. burkenroadi*. We examined type material of *B. burkenroadi* and found the thelycum and petasma of this species to be greatly elaborate and structurally similar to those seen in the core species of *Gennadas* (figures 3*d* and 5*d*), and significantly different from those in *Bentheogennema*. Our phylogenetic analysis showed that this species is deeply nested into the *Gennadas* tree and belongs to *G. brevirostris* species group. Hence, *B. burkenroadi* belongs to *Gennadas* which makes some changes in the diagnosis of *Gennadas* necessary:

*Gennadas* Bate, 1881.

*Emended diagnosis*: Integument membranous; rostrum not reaching eye cornea, armed with one apical and one dorsal tooth, setose in between; carapace with distinct cervical and postcervical sulci reaching dorsal midline, antennal angle rounded, branchiostegal angle square, hepatic and branchiostegal carinae weak; abdomen with first to fifth somites dorsally rounded and sixth somite dorsally carinate; telson posteriorly truncate, with a single pair of movable posterolateral spines. Eyestalks with long tubercle; first maxilliped with exopod not segmented distally; fourth and fifth pereopods with dactyl slender, entire. Petasma as wide lamina with developed elongate PE; appendix masculina bilamellate, with inner lobe spinose; thelycum elaborated, formed by sixth to eighth thoracic segments.

#### 4.1.2. New classification of *Gennadas* and new species groups

Burkenroad [4] found that in ‘all species of *Gennadas* with independent spermathecal orifices (e.g., open thelycum) the distolateral lobe of the petasma is entire, not subdivided; whereas in all species with orifices contained within a common atrium (e.g., closed thelycum), the distolateral lobe is bifurcated’. On the basis of these findings, he divided *Gennadas* into two groups, which were followed hereafter. However, this subdivision of *Gennadas* could not be further substantiated by our work. First of all, the information revealed by SEM showed that in most *Gennadas*, the thelycum is more or less closed and the orifices are covered by the shields either anteriorly or posteriorly. Open thelyca were found only in *G. incertus* and *G. propinquus* (figure 5*f*,*g*), in which the PE (the distolateral lobe) of the petasma was either divided (*G. incertus*, figure 3*f*) or entire (*G. propinquus*, 2G). Second, there are numerous exceptions from Burkenroad's rule, for example *G. barbari* (not known to Burkenroad [4]), and *G. incertus* both have shallow seminal receptacles and greatly elaborate petasma; conversely, *G. elegans*, *G. kempi*, *G. capensis*, *G. parvus* and *G. sordidus* all have deeply closed seminal receptacles and relatively simple petasma. Instead of Burkenroad's scheme, we propose a new phylogeny-based classification.

*Gennadas* consists of two major phylogenetic lineages: *G. elegans* and the rest of *Gennadas*. Contrary to the core *Gennadas* but like other Benthesicymidae (*Bentheogennema* and *Benthesicymus*), *G. elegans* has a simple petasma without LA, PM and grasping structure on PI. However, the morphology of the thelycum in *G. elegans* is elaborate and more similar to the core *Gennadas* than to the other genera. Our phylogenetic analysis did not support grouping this species with the core genus. *Gennadas elegans* probably should be placed in a separate genus *Amalopenaeus* (first description: *Amalopenaeus elegans* Smith, 1882 [21]) in order to keep the monophyly of *Gennadas*, but we are reluctant to do this until a global phylogenetic revision of the whole family Benthesicymidae is completed.

Phylogenetic analysis has shown that the core *Gennadas* further branches into four very robust clades, each consisting of two to five species. To better represent the phylogenetic information in the classification, we subdivide *Gennadas* into four species groups and treat *G. elegans* as separate from these.

G. elegans

*Diagnosis*: (figures 2*a* and 4*a*)

Thelycum: specialized setae absent; sixth sternite with posteriorly directed scutum in the anterior part and with W-shaped posterior prominence; seventh sternite undivided, as a simple oval plate.

Petasma: PI without distal grasping structure, LA and PM absent, PE leaf-like. Both lobes of appendix masculina spinose.

*G. valens* species group

*Diagnosis*: (figures 2*g*,*h* and 4*g*,*h*)

Thelycum: specialized setae present; sixth sternite simple subtriangular, without scutum; seventh sternite divided into two shields, anterior shield bat-like, lateral edges not reaching coxae, posterior shield trapezoid, with anterior incision, lateral edges not reaching coxae, anteriorly armed with a row of strong spines; eighth sternite with median groove in the posterior part, without scutum.

Petasma: PI with distal grasping structure, LA long and distally extended, directed nearly orthogonal to main lamina, PM apically cleft into smaller interior and larger exterior lobules, PE with rounded apical notch as bit of key. Outer lobe of appendix masculina unarmed.

Species included: *G. talismani* and *G. valens.*

*G. brevirostris* species group

*Diagnosis*: (figures 3*c*,*d*,*g*,*h* and 5*c*,*d*,*g*,*h*)

Thelycum: specialized setae present; sixth sternite simple subtriangular; seventh sternite divided into two shields, posterior shield as long chitinized strip, laterally produced beyond coxae, with two groups of lateral setae in addition to two groups of sublateral setae.

Petasma: PI with distal grasping structure, LA, PM and PE present. Outer lobe of appendix masculina unarmed.

Species included: *G. brevirostris*, *G. burkenroadi*, *G. propinquus* and *G. scutatus.*

*G. bouvieri* species group

*Diagnosis*: (figures 3*a*,*b*,*e*,*f* and 5*a*,*b*,*e*,*f*)

Thelycum: specialized setae present; sixth sternite simple subtriangular; seventh sternite divided into two parts, both simple and unspecialized, without prominent relief, posterior part as soft plain narrow strip.

Petasma: PI with distal grasping structure, LA, PM and PE present. Outer lobe of appendix masculina unarmed.

Species included: with five species: *G. bouvieri*, *G. capensis*, *G. incertus* and *G. kempi.*

*G. parvus* species group

*Diagnosis*: (figures 2*b–f* and 4*b–f*)

Thelycum: specialized setae present; sixth sternite simple subtriangular; seventh sternite divided into two shields, anterior shield bilobed, with medial depression, lateral edges not reaching coxae, posterior shield subtriangular, with beak-like anterior elevation, lateral edges not reaching coxae.

Petasma: PI with distal grasping structure, LA, PM and PE present. Outer lobe of appendix masculina unarmed.

Species included: with five species: *G. barbari*, *G. gilchristi*, *G. parvus*, *G. sordidus* and *G. tinayrei.*

#### 4.1.3. Key to genera of Benthesicymidae and key species of *Gennadas*

Key to Genera of Benthesicymidae

1. Telson armed with a single pair of terminal spines  — *Gennadas* Spence Bate, 1881
  — Telson armed with lateral spines in addition to terminal spines  — 2
2. Fifth abdominal somite without dorsal carina  — *Bentheogennema* Burkenroad, 1936
  — Fifth abdominal somite with dorsal carina on the posterior part at least  — 3
3. Dactyli of fourth and fifth pereopods greatly elongate and subsegmented
    — *Benthonectes* Smith, 1885
  — Dactyli of fourth and fifth pereopods ordinary, unisegmented  — 4
4. Rostrum dorsally unarmed or bearing a single rudimentary dorsal tooth. Third abdominal segment with well-developed dorsal carina. Petasma: PE greatly overreaching PI
    — *Altelatipes* Crosnier & Vereshchaka, 2008
  — Rostrum dorsally armed with one or more well-developed dorsal teeth. Third abdominal segment without well-developed dorsal carina. Petasma: PE not overreaching PI
    — *Benthesicymus* Spence Bate, 1881

Key to Species of *Gennadas*

**Figure 7.**
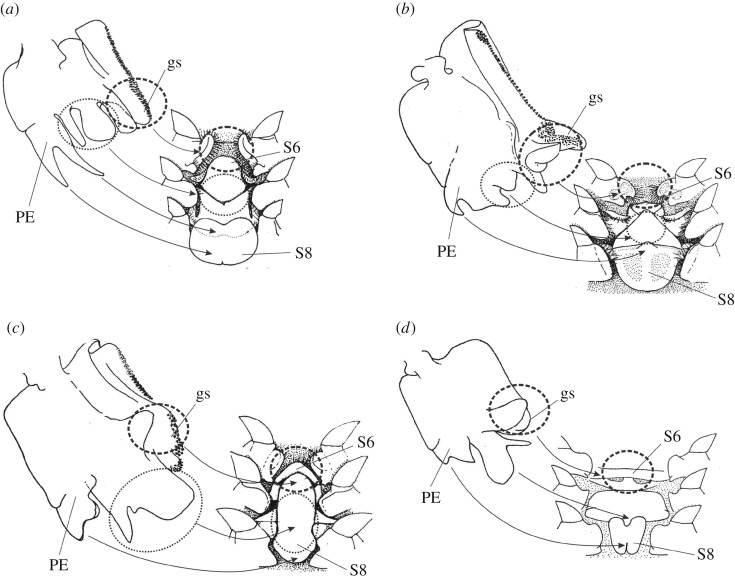
Possible position of the petasma and the thelycum during copulation to show how characters fit to each other: *G. incertus* (*a*), *G. parvus* (*b*), *G. scutatus* (*c*) and *G. talismani* (*d*). We illustrate right halves of the semitransparent petasmata to show the relief of the opposite side faced to the thelyca. Abbreviations: PE, pars externa; gs, grasping structure; S6, sixth thoracic sternite; S8, eighth thoracic sternite.

1. Petasma: PI without distal grasping structure; LA and PM absent (figure 2*a*); both lobes of appendix masculina spinose. Thelycum: S6 with posteriorly directed scutum in the anterior part and with W-shaped posterior prominence; S7 undivided, as a simple oval plate (figure 4*a*)
    — *G. elegans* (Smith, 1882)
  — Petasma: PI with distal grasping structure; LA and PM present; outer lobe of appendix masculina unarmed. Thelycum: S6 simple subtriangular; S7 divided, not as a simple oval plate
    — 2
2. Petasma: LA club-like; PE with a rounded apical notch as a bit of a key (figure 2*g*,*h*). Thelycum: PS7 trapezoid, with anterior incision, lateral edges not reaching coxae, anteriorly armed with a row of strong spines; S8 with median groove in the posterior part (figure 4*g*,*h*)
    — 3 (*G. valens* species group)
  — Petasma: LA not club-like; PE not as a bit of a key. Thelycum: PS7 subtriangular or W-shaped, or as a linear strip; S8 without median groove in the posterior part  — 4
3. Petasma: PM with lobules subequal in width (figure 2*g*). Thelycum: S8 anteriorly convex, unarmed (figure 4*g*)— *Gennadas valens* (Smith, 1884)
  — Petasma: PM with lobules unequal in width (figure 2*h*). Thelycum: S8 anteriorly bilobe, with two spinose projections (figure 4*h*)— *Gennadas talismani* Bouvier, 1906
4. Thelycum: PS7 as a narrow chitinized strip, produced laterally beyond coxae, with two groups of lateral setae in addition to two groups of sublateral setae (figure 5*c*,*d*,*g*,*h*)
    — 5 (*G. brevirostris* species group)
  — Thelycum: PS7, if narrow, not chitinized, not produced laterally beyond coxae, with two groups of sublateral setae only  — 8
5. Petasma: LA laterally expanded, overlapping PI in the lateral direction; PE narrow (figure 3*c*,*d*). Thelycum: AS7 as two separate lateral ear-like structures; PS7 as a W-shaped strip; S8 with short anteriorly spinose scutum (figure 5*c*,*d*)— 6
  — Petasma: LA not overlapping PI in the lateral direction; PE wide (figure 3*g*,*h*). Thelycum: AS7 W-like, with lateral edges reaching coxae; PS7 as a nearly linear strip; scutum of S8, if present, not short and spinose (figure 5*g*,*h*)— 7
6. Petasma: LA without hooks; PM divided; PE unarmed (figure 3*c*). Thelycum: S6 with scutum directed posteriorly; S8 with scutum concave, armed with uninterrupted row of spines (figure 5*c*)
    — *Gennadas brevirostris* Bouvier, 1905
  — Petasma: LA with an apical hook, PM entire; PE serrate (figure 3*d*). Thelycum: S6 with scutum directed anteriorly; S8 with scutum truncate, armed with an interrupted row of spines (figure 5*d*)
    — *Gennadas burkenroadi* (Krygier, Wasmer, 1975)
7. Petasma: PE entire (figure 3*g*). Thelycum: S8 without scutum (figure 5*g*)
    — *Gennadas propinquus* Rathbun, 1906
  — Petasma: PE apically cleft (figure 3*h*). Thelycum: S8 with a long unarmed scutum (figure 5*h*)
    — *Gennadas scutatus* Bouvier, 1906
8. Thelycum: PS7 plain, without prominent relief, as an unchitinized narrow strip (figure 5*a*,*b*,*e*,*f*)
    — 9 (*G. bouvieri* species group)
  — Thelycum: PS7 with prominent relief, subtriangular (figure 4*b–f*)
    — 12 (*G. parvus* species group)
9. Petasma: LA soft; PM nearly reaching the end of PI (figure 3*a*,*b*). Thelycum: AS7 chitinized, subrectangular, with a pair of lateral locks overlapping scutum (figure 5*a*,*b*)— 10
  — Petasma: LA rigid; PM not reaching the end of PI (figure 3*e*,*f*). Thelycum: AS7 unchitinized, as plain strip, without lateral locks overlapping scutum (figure 5*e*,*f*)— 11
10. Petasma: PM and PE bifid (figure 3*a*). Thelycum: S6 with large posteriorly extended scutum in the posterior part (overlapping AS7 and PS7); S8 with a pair of anterolateral spinose extensions (figure 5*a*) — *Gennadas bouvieri* Kemp, 1909
  — Petasma: PM and PE entire (figure 3*b*). Thelycum: S6 without scutum; S8 without spinose extensions (figure 5*b*)— *Gennadas kempi* Stebbing, 1914
11. Petasma: grasping structure greatly enlarged, inflated; PM entire; PE shallowly notched (figure 3*e*). Thelycum: S6 with scutum directed posteriorly; S8 without shield (figure 5*e*)
    — *Gennadas capensis* Calman, 1925
  — Petasma: grasping structure small, not inflated; both PM and PE deeply cleft (figure 3*f*).
  Thelycum: S6 with scutum directed anteriorly; S8 with a large oval shield (figure 5*f*)
    — *Gennadas incertus* (Balss, 1927)
12. Petasma: PT with a greatly expanded apron-like apical structure (figure 2*e*,*f*). Thelycum: beak-like structures on S6 and AS7 forming forceps present; S8 without shield (figure 4*e*,*f*)
    — 13
  — Petasma: PT without greatly expanded apron-like apical structure (figure 2*b–d*). Thelycum: no beak-like structures on S6 and AS7 not forming forceps; S8 with anterior shield (figure 4*b–d*)
    — 14
13. Petasma: LA with apron-like apical structure apically concave (figure 2*e*). Thelycum: AS7 with a pair of spinose lateral protuberances at base of fourth pereopods (figure 4*e*)
    — *Gennadas parvus* Spence Bate, 1881
  — Petasma: LA with apron-like apical structure apically convex (figure 2*f*). Thelycum: AS7 without spinose lateral protuberances at base of fourth pereopods (figure 4*f*)
    — *Gennadas sordidus* Kemp, 1910
14. Petasma: PE deeply cleft (figure 2*b*). Thelycum: AS7 without shield; S8 with anteriorly setose shield (figure 4*b*)— *Gennadas barbari* Vereshchaka, 1990
  — Petasma: PE shallowly notched (figure 2*c*,*d*). Thelycum: AS7 with anteriorly directed bilobed shield; shield on S8 not setose (figure 4*c*,*d*)— 15
15. Petasma: PM divided; PE unarmed (figure 2*c*). Thelycum: no setose medial processus extending posteriorly from the fifth thoracic segment; scutum of S8 anteriorly concave (figure 4*c*)
    — *Gennadas gilchristi* Calman, 1925
  — Petasma: PM entire; PE with lateral lobule serrate (figure 2*d*). Thelycum: fifth thoracic segment with setose medial processus extending posteriorly; scutum of S8 anteriorly convex (figure 4*d*)
    — *Gennadas tinayrei* Bouvier, 1906

### 4.2. Phylogenetic implications

The phylogenetic tree shows that the medium-level clades (the species groups) are primarily supported by thelycum-related characters, while petasma-related characters are more important for the support of the terminal clades. The greater importance of the thelycum for the main branches of the tree is also illustrated when comparing the thelycum-based and the petasma-based MP trees: the female tree is much more resolved than the male tree and has the same topology as the ‘total evidence’ tree. It is remarkable that the terminal clades (species pairs) are similar in all trees. However, the clade support is weaker in the male tree, where some species group-level clades fail to appear. We here hypothesize that the thelycum-based characters were first to appear in evolution followed by the petasma-based characters. Among these possibly co-evolving characters are the structure of the sixth and seventh thoracic sternites and the presence of specialized groups of setae on thelycum.

Other thelycum-related characters are much less conservative. Even such remarkable species-diagnostic characters as scuti, occurring in different parts of the thelycum (from S6 to S8) and being very characteristic in shape, size and armature, seem evolved later in evolution, at the species level. In most species, they are not fully homologous, which is indicated by the fact that they occur on different somites.

The petasma is generally an evolutionary more plastic character than the thelycum. Only presence of the major lobes (LA, PM and the grasping structure of PI) determining the rough topology of the petasma is conservative. Such characters as further division and shape of the lobes, the presence of additional lobules and their serration, etc. are species-specific and have appeared late.

We thus suggest a ‘lock and key’ hypothesis and tentatively conclude that females of *Gennadas* were the first to evolve elaborate sexual structures externally (topology of sternites) later followed by males, in which a very specific petasma is present in each species. The lobules of the petasma, which are very specific in size and shape, probably evolved to fit species-specific parts of the thelycum (scuti and microrelief).

### 4.3. Coevolution of characters and functional morphology of copulation

An analysis of the coevolution between central aspects of the sexual structures showed a couple of statistically significant correlations: (i) between the external part of the petasma and the posterior part of the thelycum and (ii) between the internal and medial parts of the petasma and the anterior part of the thelycum.

The explanation for the coevolution between certain sexual structures in females and males must be that these structures interact functionally during copulation. However, *in situ* observations of copulation in Dendrobranchiata are very rare for coastal species and entirely absent for pelagic ones. Bauer [22] was among the first to observe and analyse copulation of the penaeid shrimp *Sicyonia dorsalis*. He found that the position of the male was at right angles below the female during copulations and that males were able to inseminate only the spermatheca on one side per successful copulation. He proposed that the petasma is not a sperm injection device; instead, this organ is used to hook onto the female thelycum, adjusting the proper position during copulation.

Assuming that a comparable mechanism exists in pelagic *Gennadas*, we reconstruct the details of spermatophore deposition based on which parts of the petasma and thelycum fit to each other. Figure 7 shows a hypothesized fit between male and female copulatory structures during copulation for four species representing each of the four species groups of the core *Gennadas*. It is clear that in all cases, PE of the petasma always fits to S8 of the thelycum, and that the grasping structure of PI of the petasma fits to the seminal receptacles of the thelycum. The grasping structure, which is synapomorphic for the core *Gennadas*, may serve for carrying the spermatophore and fixing it to the receptacles. It is noteworthy that the nearly terminal position of the grasping structure on the petasma facilitates receiving the spermatophores from the genital apertures, which face the dorsal (anterior) side of the petasma.

In some species, the petasma and the thelycum both have very peculiar structures, which provide further evidence for how female and male structures fit with each other during copulation. For example, *G. incertus* (figure 7*a*) has an unusual fork-like PE of the petasma, which may be passed under the cap of the mushroom-like structure on S8 of thelycum (which is also a unique structure). The apron-like apex of LA in the petasma of *G. parvus* is suitable for hanging onto the coxa of the third pereopod (figure 7*b*). The mitten-like PM of the petasma of *G. scutatus* and *G. talismani* may be adapted to pass under the scutum of the former and anterior setose projections of S8 of the latter (figure 7*c*,*d*).

### 4.4. Ecological and biogeographical implications

Recent analyses have suggested that the elaborate male petasmata are important for successful colonization of the pelagic zone by shrimp-like eucarids [23]. In the turbulent and hydrographically dynamic pelagic zone, successful copulation depends on perfect fixation and possible stimulation of mates during spermatophore transfer and thus on the presence of suitable copulatory structures. In taxa such as Sergestidae, Luciferidae and Euphausiidae, the petasma is very elaborate consisting of branches with numerous lobi and processi, probably among the most elaborate in the animal kingdom. Surprisingly, the thelyca in the same families are simple and not greatly specialized. By contrast, the core *Gennadas* of Benthesicymidae is characterized by a simpler (but still elaborate) petasma and by the most elaborate thelycum among the eucarids. The thelyca of *Gennadas* species play a larger role in stable copulation and sperm transfer than in other eucarids.

*Gennadas* is among the most widely distributed pelagic genera. Indeed, four species (25%) are panoceanic: *G. bouvieri*, *G. capensis*, *G. scutatus* and *G. tinayrei*. Four species live in the Atlantic (*G. brevirostris*, *G. elegans*, *G. talismani* and *G. valens*), four species live in the Indo-Pacific (*G. incertus*, *G. parvus*, *G. propinquus* and *G. sordidus*) and two species live in the Indo-West Pacific (*G. gilchristi* and *G. kempi*). There are only two species with regional distribution: *G. burkenroadi* from the northeast Pacific and *G. barbari* from the southeast Pacific. The latter is benthopelagic and associated with seamounts of the Nazca and Sala-y-Gomez Ridges, which may explain its restricted distribution [3,10].

The greatly elaborate copulatory structures of *Gennadas* and the absence of significant individual variations in these structures both favour sexual isolation between species, which are otherwise ecologically similar in the mesopelagic habitat.

## Supplementary Material

Examined material

## Supplementary Material

List of characters and their coding

## Supplementary Material

Data matrix

## Data accessibility

Our data are deposited at the Dryad Digital Repository: http://dx.doi.org/10.5061/dryad.v4d24 [24].
