# Supplementary material for: The genus Gennadas (Benthesicymidae: Decapoda): morphology of copulatory characters, phylogeny and coevolution of genital structures
Source: R Soc Open Sci. 2017 Dec 6;4(12):171288. doi: 10.1098/rsos.171288 (PMC5750024; doi:10.1098/rsos.171288)
Supplement: Examined material [file rsos171288supp1.doc]

Appendix 1. Examined material. NHM, British Museum (Natural History); NMNH, National Museum of Natural History, Paris; USNM, United States National Museum; YPM, Yale Peabody Museum; ZMUC, Zoological Museum, University of Copenhagen.

| **Species** | **Location** |
| --- | --- |
| *Aristaeomorpha foliacea* | ZMUC 4451 |
| *Bentheogennema burkenroadi* | USNM 150841; Sta.: MT 2130, North Pacific Ocean, United States, Off Oregon; Centroid: 44 36 N, 125 50 W, Date Coll.: 1 Mar 1973, Depth: 0-600 M. Acc.: 312431 |
| *Bentheogennema burkenroadi* | USNM 150837; Sta.: MT604; North Pacific Ocean, United States, Off Oregon; Centroid: 44 40 N, 130 10 W; Date coll. 25 Feb 1965, Depth: 0-200 m, Acc.: 312431. |
| *Bentheogennema borealis* | NMNH-IU-2016-9205 RV Tansei-Maru, KT-02 cruise, ST-IK-1, 28°03,95’N, 130°31.89’E, 0-2000 m, IKMT-net. 15-04.2002. |
| *Bentheogennema intermedia* | NMNHMP-NA 12445. ABYPLANE St CP14, 39°59,1’N - 15°00,2’W, chaultage 5330 m. 8.06.1981, 14:55 – 16:00. |
| *Bentheogennema pasithea* | ZMUC Mombasa-Seyshelles, 3°38'S, 52°43 E, Depth 4700-4970; TOT. 27.3.1951, Galathea, 266 |
| *Bentheogennema stephenseni* | MNHN-Na-6453. “Marion Dufresne”, SAFARI II. Central Indian Ocean, St. CP 10, 2.08.1981. 01°43’N, 87°08’E, Prof. 4350 m. |
| *Bentheogennema stephenseni* | MNHN, NOUVELLE-CALEDONIE: Ride de Norfolk, N/O “Tangaroa” campagne HALIPRO 2, Stn BT85, 935-1100m, 23°40’S, 168°05’E. 23.11.1996 |
| *Gennadas barbari* | ZMUC RV «Vityaz», 17 cruise, St. 1985 |
| *Gennadas bouvieri* | ZMUC RV «Vityaz», 17 cruise St. 2785 |
| *Gennadas bouvieri* | ZMUC RV «Vityaz», 17 cruise,St. 2604 |
| *Gennadas bouvieri* | ZMUC RV «Vityaz», 17 cruise,St. 2785 |
| *Gennadas bouvieri* | ZMUC RV «Vityaz», 17 cruise, St. 2578 |
| *Gennadas bouvieri* | ZMUC RV «Logatchev», 37 cruise St 112 РТАК |
| *Gennadas brevirostris* | ZMUC “Thor” 1910, St.234 |
| *Gennadas brevirostris* | ZMUC "Thor" St.65, 1300 m, 35°53' N, 7°21'W. 24.2.1909 160 mw |
| *Gennadas brevirostris* | ZMUC "Thor" St.232, 3760 m, 36°28' N, 9°5'W. 9.9.1910 300 mw |
| *Gennadas brevirostris* | ZMUC Dana 1930, St. 4149(4) 11.6.1930 |
| *Gennadas capensis* | ZMUC RV «Vityaz», 17 cruise St. 2642 |
| *Gennadas capensis* | ZMUC RV «Vityaz», 17 cruise, St. 2604 |
| *Gennadas capensis* | ZMUC RV «Logatchev», 37 cruise, St.99 IKMT PTAK |
| *Gennadas capensis* | ZMUC RV «Vityaz», 17 cruise, St. 2785 |
| *Gennadas crassus* | NHM 1958.6.3.12 holotype, St.121, 5°39'00 S,39°38'30”E, 21.02.1934, 570 fms. Indian Ocean (E. African) Coll. John Murray Expd, 1933-34. Det. N. Tirmizi |
| *Gennadas elegans* | ZMUC Dana 1930, St.4139 (3) |
| *Gennadas elegans* | ZMUC Dana 1930, St 4017 (1)27.3.1930 29°11' N, 14°14'W M.W. 5000 |
| *Gennadas gilchristi* | ZMUC RV «Vityaz», 17 cruise, St. 2675 |
| *Gennadas gilchristi* | ZMUC RV «Vityaz», 17 cruise, St. 2642 |
| *Gennadas gilchristi* | ZMUC RV «Vityaz», 17 cruise, St. 2714 |
| *Gennadas incertus* | ZMUC RV «Vityaz», 17 cruise,St. 2604 |
| *Gennadas* incertus | ZMUC RV «Vityaz», 17 cruise, St. 2789 |
| *Gennadas incertus* | ZMUC RV «Vityaz», 17 cruise, St. 2604 |
| *Gennadas kempi* | ZMUC Galathea Exp, 1950-52, St.200, 29°39'S, 37°01'E, depth 5110, 18.2.1951 |
| *Gennadas kempi* | ZMUC Galathea Exp. 1950-1952, St. 200, Off Natal. HOT. 29°39' S,37°01'E, Depth 5100 m, 18.2.1951, 21-55. |
| *Gennadas parvus* | ZMUC RV «Vityaz», 17 cruise St. 2788 |
| *Gennadas parvus* | ZMUC RV «Vityaz», 17 cruise, St. 2604 |
| *Gennadas parvus* | ZMUC RV «Vityaz», 17 cruise, St. 2675 |
| *Gennadas parvus* | ZMUC Dana 1929, St.3919(2), 8.12.29 |
| *Gennadas propinquus* | ZMUC RV «Vityaz», 17 cruise St. 2789 |
| *Gennadas propinquus* | ZMUC RV «Vityaz», 17 cruise, St. 2604 |
| *Gennadas scutatus* | ZMUC RV «Vityaz», 17 cruise, St. 2604 |
| *Gennadas scutatus* | ZMUC RV «Vityaz», 17 cruise, St. 2578 |
| *Gennadas sordidus* | ZMUC RV «Vityaz», 17 cruise St. 2578 |
| *Gennadas talismani* | ZMUC Dana 1930, St. 3999-2. 3°45’S, 10°00’W, mw 600, S 200 |
| *Gennadas talismani* | ZMUC Dana 1930, St. 3999-2, |
| *Gennadas tinayrei* | ZMUC RV «Vityaz», 17 cruise,St. 2578 |
| *Gennadas tinayrei* | ZMUC RV «Vityaz», 17 cruise, St. 2777 |
| *Gennadas valens* | ZMUC RV «Logatchev», 37 cruise, St. 112 РТАК |
| *Gennadas vallens* | ZMUC "Thor" St. 65, 1300 m, 35°53’N, 7°21’W, 24.2.1909, 1600 mw |
