# Supplementary material for: The genus Gennadas (Benthesicymidae: Decapoda): morphology of copulatory characters, phylogeny and coevolution of genital structures
Source: R Soc Open Sci. 2017 Dec 6;4(12):171288. doi: 10.1098/rsos.171288 (PMC5750024; doi:10.1098/rsos.171288)
Supplement: List of characters and their coding [file rsos171288supp2.doc]

Appendix 2. List of characters and their coding. Abbreviations, thelycum: S6 – sternite of the sixth thoracic segment; AS7 and PS7 – anterior and posterior parts of sternite of the seventh thoracic segment; S8 – sternite of the eight thoracic segment. Petasma: PI − pars interna; LA − lobus accessourius; PM − pars media, PE − pars externa.

| Character No | Character state | State No |
| --- | --- | --- |
|  | **THELICUM** |  |
| 0 | Thelicum and adjacent coxae bearing setae only | 0 |
|  | Thelicum and adjacent coxae bearing groups of specialized stout spines in addition to setae | 1 |
| 1 | S6, posterior subtriangular/trapezoid prominence: absent | 0 |
|  | S6, posterior subtriangular/trapezoid prominence: present | 1 |
| 2 | S6, posterior W-shaped prominence: absent | 0 |
|  | S6, posterior W-shaped prominence: present | 1 |
| 3 | S6, medial group of setae: absent | 0 |
|  | S6, medial group of setae: present | 1 |
| 4 | S6, posteriorly directed scutum in anterior part: absent | 0 |
|  | S6, posteriorly directed scutum in anterior part: present | 1 |
| 5 | S6, large posteriorly extended scutum in posterior part (overlapping AS7 and PS7): absent | 0 |
|  | S6, large posteriorly extended scutum in posterior part (overlapping AS7 and PS7): present | 1 |
| 6 | S6, beak-like posterior prominence directed posteriorly: absent | 0 |
|  | S6, beak-like posterior prominence directed posteriorly: present | 1 |
| 7 | S6, small flat posteriorly directed scutum in posterior part: absent | 0 |
|  | S6, small flat posteriorly directed scutum in posterior part: present | 1 |
| 8 | S6, anteriorly directed scutum in posterior part: absent | 0 |
|  | S6, anteriorly directed scutum in posterior part: present | 1 |
| 9 | Beak-like structures on S6 and AS7 forming a forceps: absent | 0 |
|  | Beak-like structures on S6 and AS7 forming a forceps: present | 1 |
| 10 | Sternite 7: undivided | 0 |
|  | Sternite 7: divided into two shields | 1 |
| 11 | Sternite 7 as simple oval plate: absent | 0 |
|  | Sternite 7 as simple oval plate: present | 1 |
| 12 | AS7: with prominent relief | 0 |
|  | AS7: without prominent relief | 1 |
| 13 | AS7: chitinized | 0 |
|  | AS7: not chitinized | 1 |
| 14 | AS7 plain and chitinized: with prominent relief or unchitinized | 0 |
|  | AS7 plain and chitinized: plain and chitinized | 1 |
| 15 | AS7: polygonal | 0 |
|  | AS7: as plain strip | 1 |
| 16 | AS7 plain subrectangular: absent | 0 |
|  | AS7 plain subrectangular: present | 1 |
| 17 | AS7 trapezoid, with posterior incision: absent | 0 |
|  | AS7 trapezoid, with posterior incision: present | 1 |
| 18 | AS7 anteriorly entire | 0 |
|  | AS7 anteriorly bilobed | 1 |
| 19 | AS7 bat-like, lateral edges not reaching coxae: absent | 0 |
|  | AS7 bat-like, lateral edges not reaching coxae: present | 1 |
| 20 | AS7 W-like, lateral edges reaching coxae: absent | 0 |
|  | AS7 W-like, lateral edges reaching coxae: present | 1 |
| 21 | AS7 entire | 0 |
|  | AS7 as two separate lateral ear-like structures | 1 |
| 22 | AS7, anderiorly directed notched elevation: absent | 0 |
|  | AS7, anderiorly directed notched elevation: present | 1 |
| 23 | AS7, medial depression: absent | 0 |
|  | AS7, medial depression: present | 1 |
| 24 | AS7, a pair of lateral protuberances at the base of coxae of third pereopod: absent | 0 |
|  | AS7, a pair of lateral protuberances at the base of coxae of third pereopod: present | 1 |
| 25 | AS7, a pair of lateral locks overlapping scutum: absent | 0 |
|  | AS7, a pair of lateral locks overlapping scutum: present | 1 |
| 26 | PS7 with prominent relief | 0 |
|  | PS7 plain, without prominent relief | 1 |
| 27 | PS7 polygonal | 0 |
|  | PS7 as an unchitinized narrow strip | 1 |
| 28 | PS7 subtriangular: absent | 0 |
|  | PS7 subtriangular: present | 1 |
| 29 | PS7, anterior part extended and overlapping medial depression of AS7: absent | 0 |
|  | PS7, anterior part extended and overlapping medial depression of AS7: present | 1 |
| 30 | PS7 trapezoid with anterior incision: absent | 0 |
|  | PS7 trapezoid with anterior incision: present | 1 |
| 31 | PS7 as long chitinized strip: absent | 0 |
|  | PS7 as long chitinized strip: present | 1 |
| 32 | PS7 as nearly straight chitinized strip: absent | 0 |
|  | PS7 as nearly straight chitinized strip: present | 1 |
| 33 | PS7 as W-shaped strip: absent | 0 |
|  | PS7 as W-shaped strip: present | 1 |
| 34 | PS7: not laterally produced beyond coxae | 0 |
|  | PS7: laterally produced beyond coxae | 1 |
| 35 | PS7, beak-like anterior elevation: absent | 0 |
|  | PS7, beak-like anterior elevation: present | 1 |
| 36 | PS7, two groups of sublateral setae: absent | 0 |
|  | PS7, two groups of sublateral setae: present | 1 |
| 37 | PS7, two groups of lateral setae in addition to two groups of sublateral setae:  absent | 0 |
|  | PS7, two groups of lateral setae in addition to two groups of sublateral setae:  present | 1 |
| 38 | PS7: anteriorly unarmed | 0 |
|  | PS7: anteriorly armed with a row of strong spines | 1 |
| 39 | PS7, scutum: absent | 0 |
|  | PS7, scutum: present | 1 |
| 40 | S8: chitinized | 0 |
|  | S8: not chitinized | 1 |
| 41 | S8, medial keel in anterior part: absent | 0 |
|  | S8, medial keel in anterior part: present | 1 |
| 42 | S8, median groove in posterior part: absent | 0 |
|  | S8, median groove in posterior part: present | 1 |
| 43 | S8, two anterolateral setose extentions: absent | 0 |
|  | S8, two anterolateral setose extensions: present | 1 |
| 44 | S8, long unarmed scutum: absent | 0 |
|  | S8, long unarmed scutum: present | 1 |
| 45 | S8, short unarmed scutum: absent | 0 |
|  | S8, short unarmed scutum: present | 1 |
| 46 | S8, long anteriorly spinose scutum: absent | 0 |
|  | S8, long anteriorly spinose scutum: present | 1 |
| 47 | S8, short anteriorly spinose scutum: absent | 0 |
|  | S8, short anteriorly spinose scutum: present | 1 |
| 48 | S8, dish-like chitinized structure above sternite: absent | 0 |
|  | S8, dish-like chitinized structure above sternite: present | 1 |
|  | **PETASMA** |  |
| 49 | PI, grasping distal structure: absent | 0 |
|  | PI, grasping distal structure: present | 1 |
| 50 | PI: rigid, erected | 0 |
|  | PI: soft, folded | 1 |
| 51 | LA: absent | 0 |
|  | LA: present | 1 |
| 52 | LA, apical apron-like structure: absent | 0 |
|  | LA, apical apron-like structure: present | 1 |
| 53 | LA, apron-like apical structure: inconspicuous or small | 0 |
|  | LA, apron-like apical structure: greatly extended | 1 |
| 54 | LA: laminar | 0 |
|  | LA: club-like, directed nearly orthogonal to main lamina | 1 |
| 55 | LA: rigid | 0 |
|  | LA: soft | 1 |
| 56 | LA: not overlapping PI in lateral direction | 0 |
|  | LA: laterally expanded, overlapping PI in lateral direction | 1 |
| 57 | PM: absent | 0 |
|  | PM: present | 1 |
| 58 | PM as short rounded lobe not reaching end of PI and PE: absent | 0 |
|  | PM as short rounded lobe not reaching end of PI and PE: present | 1 |
| 59 | PM as small anteriorly directed barb: absent | 0 |
|  | PM as small anteriorly directed barb: present | 1 |
| 60 | PM as a wide and long lobe nearly reaching end of PI and PE and directed to PI: absent | 0 |
|  | PM as a wide and long lobe nearly reaching end of PI and PE and directed to PI: present | 1 |
| 61 | PM apically cleft into larger interior and smaller exterior lobules: absent | 0 |
|  | PM apically cleft into larger interior and smaller exterior lobules: present | 1 |
| 62 | PM apically cleft into smaller interior and larger exterior lobules: absent | 0 |
|  | PM apically cleft into smaller interior and larger exterior lobules: present | 1 |
| 63 | PM as two widely separated lobes, not connected at base: absent | 0 |
|  | PM as two widely separated lobes, not connected at base: present | 1 |
| 64 | PM apically bifid, with lobules directed to each other: absent | 0 |
|  | PM apically bifid, with lobules directed to each other: present | 1 |
| 65 | PE: absent | 0 |
|  | PE: present | 1 |
| 66 | PE deeply cleft (incision between lobules as deep as incision between PE and PM): absent | 0 |
|  | PE deeply cleft (incision between lobules as deep as incision between PE and PM): present | 1 |
| 67 | PE, distal notch dividing apex into subequal branches directed slightly inward: absent | 0 |
|  | PE, distal notch dividing apex into subequal branches directed slightly inward: present | 1 |
| 68 | PE, additional small pointed lobule: absent | 0 |
|  | PE, additional small pointed lobule: present | 1 |
| 69 | PE, additional small pointed hook-like lobule: absent | 0 |
|  | PE, additional small pointed hook-like lobule: present | 1 |
| 70 | PE, additional small scoop-like lobule: absent | 0 |
|  | PE, additional small scoop-like lobule: present | 1 |
| 71 | PE, rounded apical notch as ward of key: absent | 0 |
|  | PE, rounded apical notch as ward of key: present | 1 |
| 72 | PE, narrow, with pointed apex bent inward: absent | 0 |
|  | PE, narrow, with pointed apex bent inward: present | 1 |
| 73 | PE: smooth | 0 |
|  | PE: serrate | 1 |
| 74 | PE, additional small rounded lobule: absent | 0 |
|  | PE, additional small rounded lobule: present | 1 |
|  | **TELSON** |  |
| 75 | A pair of strong terminal spines: absent | 0 |
|  | A pair of strong terminal spines: present | 1 |
| 76 | Additional strong dorsolateral spines: absent | 0 |
|  | Additional strong dorsolateral spines: present | 1 |
