# Supplementary material for: The genus Gennadas (Benthesicymidae: Decapoda): morphology of copulatory characters, phylogeny and coevolution of genital structures
Source: R Soc Open Sci. 2017 Dec 6;4(12):171288. doi: 10.1098/rsos.171288 (PMC5750024; doi:10.1098/rsos.171288)
Supplement: Data matrix [file rsos171288supp3.docx]

Appendix 3. DATA MATRIX. Missing data indicated by question marks (?); inapplicable data by hyphens (-)

Characters 0 - 52

0 5 10 15 20 25 30 35 40 45 50

| | | | | | | | | | |

Aristeomorpha foliacea 00000000000000000000000000000000000000000000000000000

Bentheogennema borealis 10000000000000000000000000000000000000000000000000000

Bentheogennema intermedia 10000000000000000000000000000000000000000000000000000

Bentheogennema pasithea 10000000000000000000000000000000000000000000000000000

Bentheogennema stephenseni 10000000000000000000000000000000000000000000000000000

Gennadas elegans 00101000000100000000000000000000000000010000000000000

Gennadas gilchristi 11000000001000000010001100001100000100000000010001010

Gennadas tynairei 11000000001000000010001100001100000100000000010001010

Gennadas barbari 11010000001000000110000100001000000110000000001001011

Gennadas parvus 11010010011000000110000110001000000110000100000001011

Gennadas sordidus 11010010011000000110000100001000000110000100000001011

Gennadas valens 11000000001000000001000000000010000010100010000001010

Gennadas talismani 11000000001000000001000000000010000010100011000001010

Gennadas bouvieri 11000100001010101000000001110000000010000000000001110

Gennadas kempi 11000000001010101000000001110000000010000000000001110

Gennadas capensis 11000000001011010000000000110000000010001000000001010

Gennadas incertus 11000000001011010000000000110000000010001000000011010

Gennadas brevirostris 11000001001000000000010000000001011011000000000101010

Gennadas burkenroadi 11000000001000000000010000000001011011000000000101010

Gennadas propinquus 11000000101000000000100000000001101011000000000001010

Gennadas scutatus 11000000101000000000100000000001101011000000100001010

Characters 53 - 76

53 58 63 68 73

| | | | |

Aristeomorpha foliacea 000000000000000000000000

Bentheogennema borealis 000000000000100000000011

Bentheogennema intermedia 000000000000000000000011

Bentheogennema pasithea 000000000000000000000011

Bentheogennema stephenseni 000000000000000000000011

Gennadas elegans 000000000000100000000010

Gennadas gilchristi 000010000000100010000010

Gennadas tynairei 000010000000100010000010

Gennadas barbari 000010000000110000000010

Gennadas parvus 100011000000100100000010

Gennadas sordidus 100011000000100100000010

Gennadas valens 010010000100100000100010

Gennadas talismani 010010000100100000100010

Gennadas bouvieri 001010010001110000000010

Gennadas kempi 001010010000100000000010

Gennadas capensis 000010100000101000000010

Gennadas incertus 000010000010101000000010

Gennadas brevirostris 000110000000100000010010

Gennadas burkenroadi 000110000000100000011110

Gennadas propinquus 000010001000100000000010

Gennadas scutatus 000010001000100001000010
